# Comparing photosynthetic characteristics of *Isoetes sinensis* Palmer under submerged and terrestrial conditions

**DOI:** 10.1038/srep17783

**Published:** 2015-12-04

**Authors:** Tao Yang, Xing Liu

**Affiliations:** 1 Laboratory of Plant Systematics and Evolutionary Biology, College of Life Science, Wuhan University, Wuhan, Hubei 430072, China

## Abstract

Crassulacean acid metabolism (CAM) is widespread in terrestrial and aquatic species, plastic in response to environmental changes. *Isoetes* L. is one of the earliest basal vascular plants and CAM is popular in this genus. *Isoetes sinensis* Palmer is an amphibious species, alternating frequently between terrestrial and aquatic environments. Given this, we investigated and compared photosynthetic characteristics over a diurnal cycle under submerged condition (SC) and terrestrial condition (TC). The results suggest that *I. sinensis* possesses a stronger CAM capacity under SC. Compared with under TC, titratable acidity levels and organic acid concentrations were more enriched under SC, whereas soluble sugar or starch and protein levels were lower under SC. Transcript analyses for nine photosynthetic genes revealed that CAM-associated genes possessed high transcripts under SC, but C_3_-related transcripts were highly expressed under TC. In addition, the enzyme activity measurements demonstrated that PEPC activity over a diurnal cycle was slightly higher under SC, whereas Rubisco activity during the daytime was greater under TC. This comprehensive study probably facilitates general understandings about the CAM photosynthetic characteristics of *Isoetes* in response to the environmental changes.

Crassulacean acid metabolism (CAM) photosynthesis is a key photosynthetic mode that C_3_ (ribulose-1, 5-carboxlyase oxygenase: Rubisco) and C_4_ (phosphoenolpyruvate carboxylase: PEPC) operate within a common cell but the two enzyme activities separate temporally^1^. In the night, nocturnal CO_2_ uptake from atmosphere or respiration is fixed by PEPC in the cytosol. A C_4_ product, malic acid is transferred to central vacuole and catalyzed by malic acid dehydrogenase (MDH). Phosphoenolpyruvate carboxylase kinase (PPCK) is also associated with CO_2_ uptake in the night. The electrochemical channel of H^+^-ATPase pump plays pivotal roles in the accumulation of malic acid in vacuole, which is activated by H^+^-translocation ATPase (V-ATPase)^2^. During the following day, the generated CO_2_ from decarboxylation of organic acids is fixed by Rubisco and further catalyzed by a multitude of enzymes in Calvin pathway, such as phosphoglycerate kinase (PGK), glyceraldehyde-3-phosphate dehydrogenase (GAPDH), phosphoribulokinase (PRK) and the others^3^,^4^. CAM photosynthesis is widespread in terrestrial and aquatic plants. It is present in approximately 16,800 species of 343 genera in 35 families^5^,^6^. CAM in aquatic vascular species has been investigated within the genera *Isoetes* L.*, Sagittaria, Crassula, Vallisneria,* and *Littorella*^7^,^8^,^9^,^10^,^11^. CAM for terrestrials is considered to respond to water-use efficiency in arid environments, whereas for aquatics, CAM is to improve competitive ability for carbon acquisition^11^. In addition, CAM is a plastic process in response to the environmental changes, such as water, pH, temperature, CO_2_, light level and ion concentrations^12^,^13^,^14^. Thus, expansive carbon fixation subcategories are observed, such as C_3_, facultative CAM, obligate CAM, latent CAM, CAM idling, and rapid-cycling CAM cycles^15^,^16^. Considering the fact that CAM acts as a CO_2_-concentration mechanism to reduce photorespiration and further elevate CO_2_ availability in terrestrial and aquatic plants, yet, carbon assimilation rate in CAM cycle is lower than C_3_ or C_4_ photosynthesis, probably owning to the dynamics of carbon fixation over a diurnal cycle^17^,^18^. Moreover, compared with C_3_, energetics and nitrogen use efficiency are not favorable choices for CAM photosynthesis^18^.

*Isoetes* is an ancient genus of heterosporous lycopsids with a unique position in plant evolution. Phylogenetic analysis shows that *Isoetes* is one of the earliest basal vascular plants, which can date back to the Devonian^19^. The genus has approximately 200 species characterized by a strongly reduced plant body^20^. Since CAM in aquatic plants was first reported in *Isoetes howellii* Engelmann^10^, most species in this genus have shown similar CAM mode regardless of a few terrestrial species, including *I. nuttallii* A. Braun ex Engelm, *I. butleri* Engelm*, I. stellenbossiensis* A.V. Duthie, and *I. durieui* Bory^9^,^10^,^11^,^21^. CAM in *Isoetes* is of great advantage to increase underwater photosynthesis at limited CO_2_ concentrations and decrease photorespiration at apparently high O_2_ concentrations^22^. In addition, typical isoetid growth form contributes to an alternative CO_2_ source from sediments. Moreover, the achlorophyllous leaf bases play important roles in elevating underwater net photosynthesis and radial O_2_ loss to the surrounding sediments^23^,^24^,^25^. Since the Paleozoic Era, the species of *Isoetes* have frequently changed growth habitats from putative amphibious to terrestrial or lacustrine transitions^11^. There is a notion that CAM pathway in modern *Isoetes* originates from seasonal wetlands and then spreads into oligotrophic lakes and terrestrial habitats^11^. To date, the genus occupies a variety of niches, including oligotrophic softwater lakes, higher-altitude wetlands, seasonal pools and intermittent streams^26^. Data in the published studies demonstrate that environmental changes have vital effects on the CAM activity in *Isoetes*^17^. It reveals that under terrestrial and semi-aquatic conditions, most species in this genus fail to produce functional stomata or loose CAM and further rely on C3 photosynthesis^15^,^17^,^27^,^28^,^29^. But quite a few species still remain CAM as the leaves are exposed to air, such as *I. karstenii, I. nuttallii* and *I. australis* Williams^11^,^22^. Furthermore, the shift from CAM to C3 mode is partially reversible, considering the fact that CAM cycle probably contributes to 40% total carbon acquisition under submerged condition (SC) but only less than 1% under terrestrial condition (TC)^12^,^17^.

*Isoetes sinensis* Palmer is an allotetraploid plant (2n = 4x = 44) distributed in East Asia^30^,^31^. *I. sinensis* is categorized as critically endangered flora on the IUCN Red List due to water pollution or eutrophication, habit degradation or loss, and competitive exclusions^32^. Additionally, *I. sinensis* is a typical amphibious plant, growing in seasonal pools and intermittent streams. The plants alternate frequently between terrestrial and aquatic environments. Previous study indicates that *I. sinensis* shares the CAM activity under SC^33^. But the photosynthetic characteristics are still unclear when exposed to TC. Moreover, little is known about the differences between the photosynthetic characteristics under TC and SC.

In this study, juvenile leaves of *I. sinensis* under TC and SC were harvested every three hours over a diurnal cycle, respectively. We used a series of experimental approaches, such as titratable acidity measurements, enzyme activity assays and quantitative real-time PCR (qPCR) analysis, to investigate the photosynthetic characteristics under TC and SC, and further compared the difference under SC with TC. Compared with under TC, the results revealed that *I. sinensis* possessed a stronger CAM capacity under SC. In addition, we identified nine photosynthetic genes and plotted a dynamic expression view based on qPCR analysis under both conditions. This comprehensive study probably facilitates general understandings about the photosynthetic characteristics of *Isoetes* in response to the changing environments and it might be instrumental for future investigations in *Isoetes*.

## Results

### Titratable acidity assay

**Figure 1:**
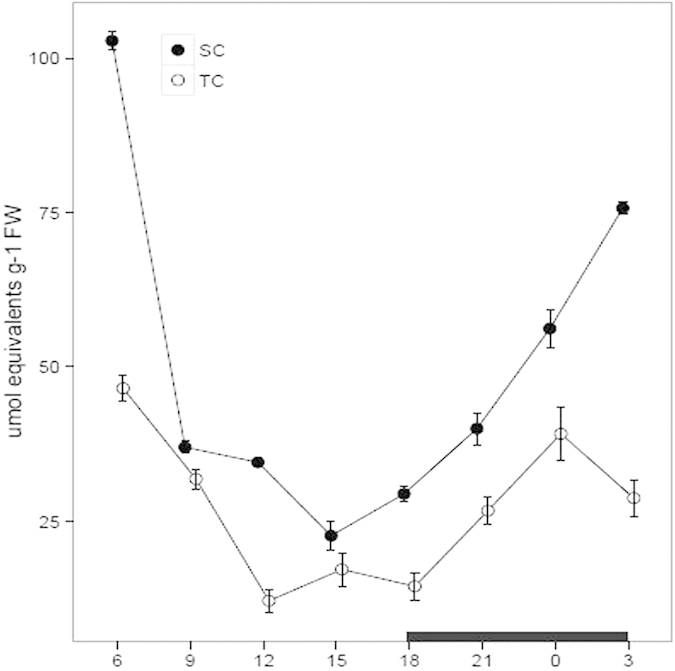
Diurnal fluctuation of titratable acidity levels under submerged (SC) and terrestrial conditions (TC). Juvenile and green leaves were harvested every three hours during the daytime (white) and night (black), under TC and SC, respectively. Each point represents the mean ± SE (n = 3).

Titratable acidity assay is considered to be one of the most popular diagnostic approaches for CAM activity^11^,^34^. In this study, titratable acidity was measured from the juvenile leaves which were harvested every three hours over a diurnal cycle under TC and SC, respectively. The acidity levels varied between 12.56 and 46.96 μmolequiv g^−1^ FW under TC and between 22.44 and 102.95 μmolequiv g^−1^ FW under SC (Fig. 1). Moreover, the acidity levels were higher under SC than TC and there was a statistically significant difference at 6, 12, 18, 0 and 3 h between the levels under SC and TC (Table 1). Under SC, the acidity levels decreased rapidly from 6 to 15 h during the daytime and increased markedly from 18 to 6 h in the night. Compared with under SC, the diurnal acidity fluctuation was lower under TC. Additionally, temperatures in water and air were recorded over a diurnal cycle, respectively. The temperature in water ranged from 14 to 23.1 °C and from 14.1 to 27.1 °C in air (Supplementary Figure S1, Table 1).

### Organic acid determination

**Figure 2:**
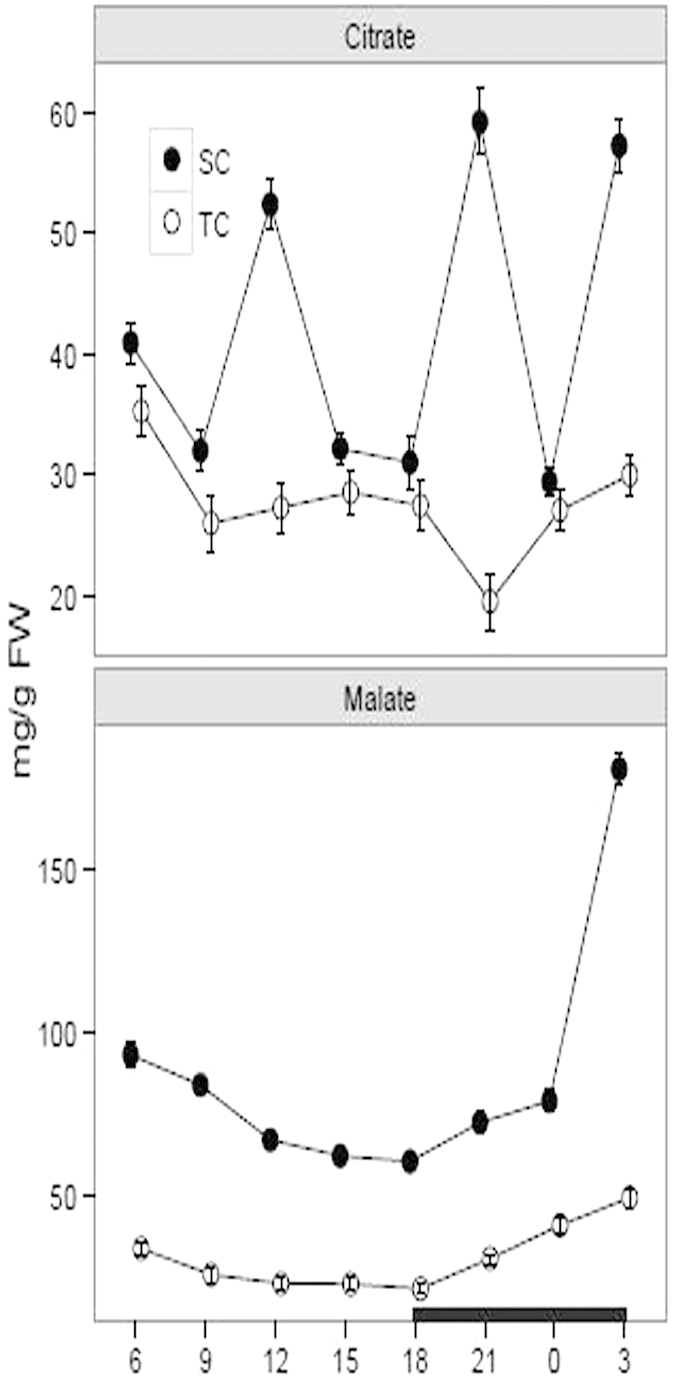
Diurnal fluctuation of malate and citrate concentrations under submerged (SC) and terrestrial conditions (TC). Juvenile and green leaves were harvested every three hours during the daytime (white) and night (black), under TC and SC, respectively. Then the malate and citrate concentrations were measured using high-performance liquid chromatograph. Each point represents the mean ± SE (n = 3).

Organic acid is another key indicator to estimate the CAM capacity^11^,^34^. To further investigate the CAM capacity under both conditions, we measured malate and citrate concentrations using high-performance liquid chromatograph (HPLC) analysis. The malate concentration varied between 60.70 and 180.93 mg. g^−1^ FW under SC and between 21.83 and 49.68 mg. g^−1^ FW under TC (Fig. 2). Compared with under TC, a markedly higher malate level was observed under SC over a diurnal cycle (Table 1). Moreover, a low amplitude during the daytime was present under both conditions, whereas in the night, the concentration obviously increased from 0 to 3 h under SC and only a slightly increased extent was occurred under TC. Similarily, the citrate concentration was higher under SC than TC and a statistically significant difference was present at 12, 21 and 3 h by comparing the concentrations under SC with TC (Fig. 2, Table 1). The citrate concentration varied between 29.44 and 52.44 mg. g^−1^ FW under SC and between 19.54 and 35.30 mg. g^−1^ FW under TC (Fig. 2).

### Starch and soluble sugar measurements

**Figure 3:**
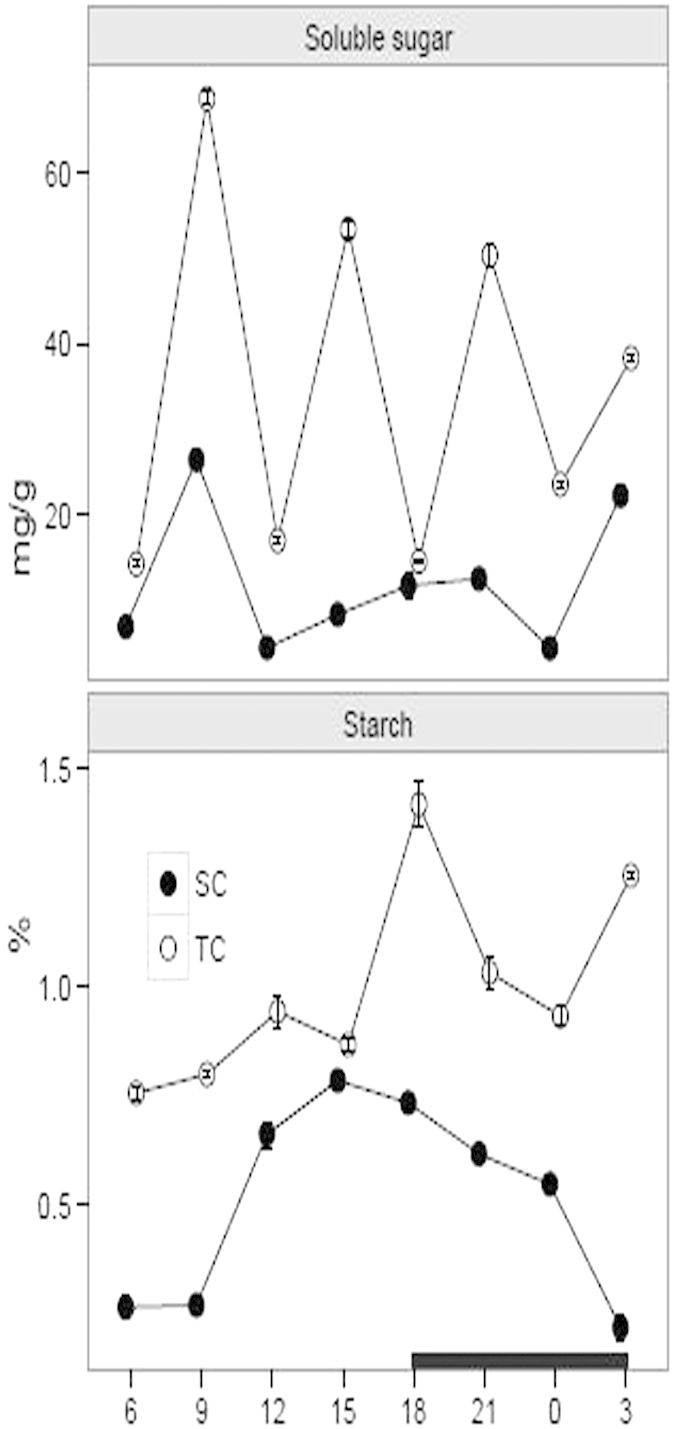
Diurnal fluctuation of soluble sugar and starch levels under submerged (SC) and terrestrial conditions (TC). Juvenile and green leaves were harvested every three hours during the daytime (white) and night (black), under TC and SC, respectively. Each point represents the mean ± SE (n = 3).

Starch and soluble sugar are considered to be vital products in the process of photosynthesis^18^,^35^. We examined the accumulation levels of soluble sugar and starch over a diurnal change under SC and TC, respectively. The result showed that the starch and soluble sugar concentrations over a diurnal cycle were consistently higher under TC than SC (Fig. 3). The starch levels in the leave tissues varied between 0.75% and 1.42% under TC and between 0.22% and 0.78% under SC. Moreover, a statistically significant difference was present at 9, 18 and 3 h by comparing the starch levels under SC with TC (Table 1). Furthermore, there was an elevated amplitude under both conditions during the daytime from 6 to 15 h, whereas a reduced fluctuation was observed throughout the night under SC and from 18 to 0 h under TC. Additionally, there was a large diurnal amplitude for the soluble sugar levels under TC, ranging from 14.23 to 68.61 mg/g (Fig. 3, Table 1).

### Soluble protein and enzyme activity

Growth under TC and SC had a statistically significant effect on the soluble protein at most of the times (Supplementary Figure 2, Table 1). Totally, the soluble protein levels were higher under TC than SC, ignoring the levels at 12 h.

**Figure 4:**
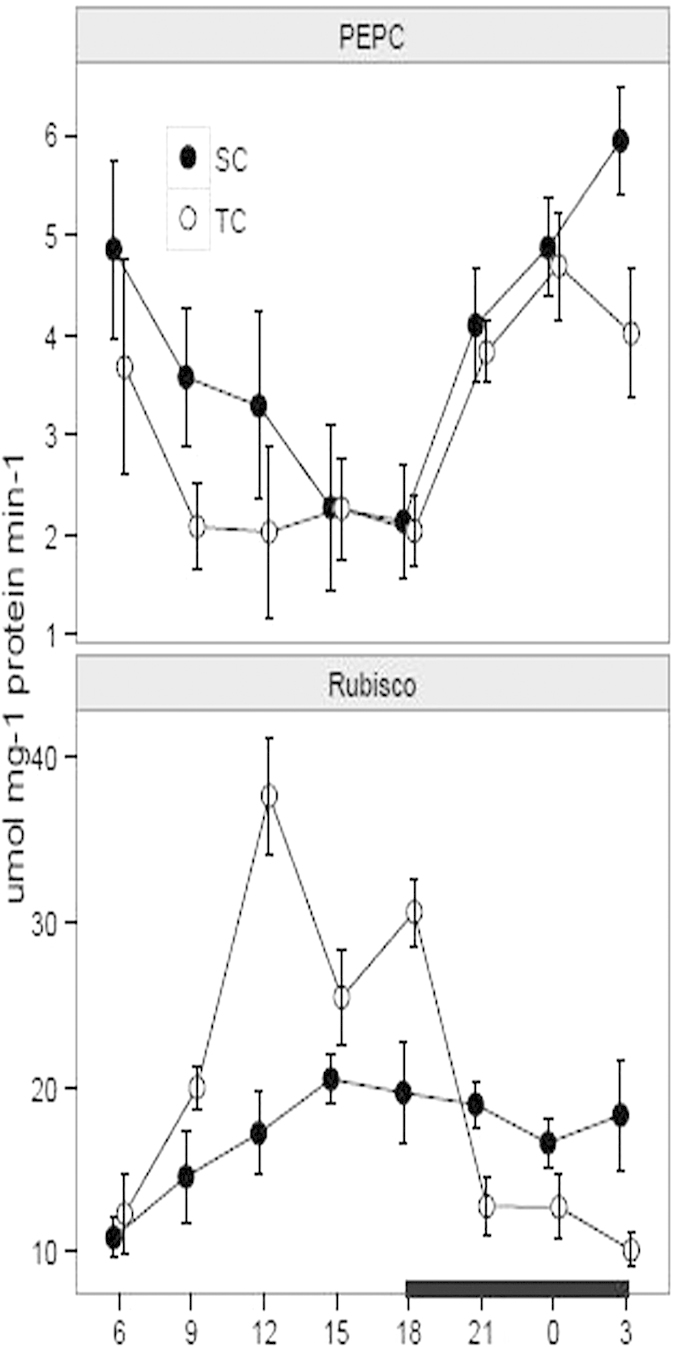
Diurnal fluctuation of enzyme activities of PEPC and Rubisco under submerged (SC) and terrestrial conditions (TC). Juvenile and green leaves were harvested every three hours during the daytime (white) and night (black), under TC and SC, respectively. Each point represents the mean ± SE (n = 3).

PEPC and Rubisco enzymes play important roles in CAM photosynthesis by operating temporary function separately within a common cell^5^,^16^. In this study, we conducted enzyme activity assay to investigate the activities of PEPC and Rubisco during a diurnal cycle under TC and SC, respectively. The PEPC activity on a protein basis varied between 2.08 and 4.69 μmol mg^−1^ protein min^−1^ under TC and between 2.19 and 5.95 μmol mg^−1^ protein min^−1^ under SC (Fig. 4). The activity under SC was slightly higher than under TC over a diurnal cycle and there was a statistically significant difference at 3, 9 and 12 h by comparing the activity under SC with TC (Table 1). Moreover, the PEPC activity consistently decreased during the daytime from 6 to 18 h and increased in the night from 18 to 0 h under both conditions, whereas the activity was decreased from 0 to 3 h under TC. Otherwise, the Rubisco activity varied between 10.01 and 37.60 μmol mg^−1^ protein min^−1^ under TC and between 10.82 and 20.72 μmol mg^−1^ protein min^−1^ under SC (Fig. 4). Compared with under SC, the Rubisco activity under TC was higher from 6 to 18 h during the daytime and lower from 21 to 3 h in the night. Furthermore, there was a statistically significant difference at 12, 18 and 3 h by comparing the activity under TC with SC (Table 1).

### Transcript determinations of photosynthetic genes using RNA-sequencing technology

We performed RNA-sequencing to determine photosynthetic genes in *Isoetes*. The sequencing datasets have been deposited in the National Center of Biotechnology Information Short Read Archive database with the accession numbers SRR1648119 under SC and SRR1646513 under TC, respectively. Out of the datasets, we totally identified nine putative homologous genes related to photosynthesis which play important roles in the process of CAM metabolism. In terms of the nine homologous genes, there were three genes related to CAM mode, including *PEPC*, *PPCK* and *MDH*; five associated with C_3_ mode, including *Rubisco*, *GADPH*, *glyceraldehyde-3-phosphate dehydrogenase (NADP+) (GAPA)*, *PRK* and *PGK*; and *V-ATPase* associated with malic acid accumulation via the electrochemical channel of tonoplast.

### Transcript accumulations of photosynthetic genes using qPCR

**Figure 5:**
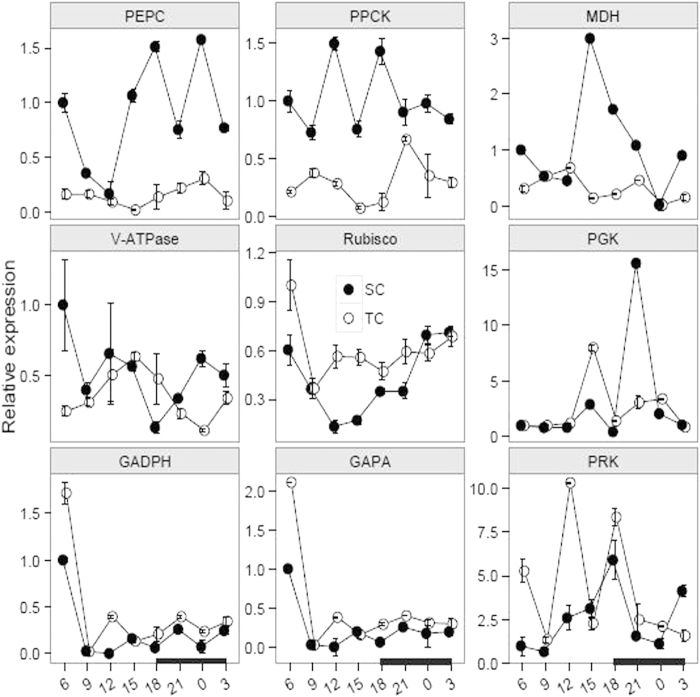
Expression levels of photosynthetic genes over a diurnal cycle under submerged (SC) and terrestrial conditions (TC). Juvenile and green leaves were harvested every three hours during the daytime (white) and night (black), under TC and SC, respectively. In total, nine photosynthetic genes were identified, including *PEPC*, *PPCK* and *MDH* for CAM mode; *Rubisco*, *GADPH*, *GAPA*, *PRK* and *PGK* for C_3_ mode; and *V-ATPase* associated with malate accumulation via the electrochemical channel of tonoplast. The plots were conducted using the ggplot2 module in R program.

We further detected the transcript accumulations by using qPCR analysis from the harvested materials under TC and SC, respectively. Totally, the results demonstrated that the *PEPC*, *PPCK* and *MDH* transcripts over a diurnal cycle were consistently higher under SC than TC (Fig. 5). For the *PEPC* transcript, the difference between the expression levels under TC and SC was statistically significant from 15 to 6 h (Table 1). For the *PPCK* and *MDH* transcripts, the diurnal expression fluctuation was larger under SC than TC, whereas the transcript difference under both conditions was statistically significant at 6, 12, 15 and 18 h for the *PPCK* transcript, and at 15 and 18 h for the *MDH* transcript (Table 1).

The accumulations of five transcripts related to C_3_ mode, including *Rubisco*, *GADPH*, *GAPA*, *PGK* and *PRK* genes, were evaluated under both conditions (Fig. 5). For the *Rubisco* gene, the transcript under SC was gradually decreasing during the daytime (from 6 to 15 h) and increasing in the night (from 18 to 3 h). Nonetheless, there was a slight increase under TC in the night and an obvious decrease from 6 to 9 h during the daytime (Table 1). In addition, the diurnal amplitude for *GAPDH* and *GAPA* transcripts resembled each other under both conditions. And the expression levels were significantly decreased from 6 to 9 h under both conditions (Table 1). Furthermore, there was a statistically significant difference at 15 and 21 h by comparing the *PGK* transcript under TC with SC (Table 1). Moreover, a large amplitude for the *PRK* transcript was occurred under both conditions and there was a statistically significant difference at 6 and 12 h by comparing the transcript under TC with SC (Table 1). In terms of the *V-ATPase* gene, the transcript under TC was increasing from 6 to 15 h and decreasing from 15 to 0 h, whereas the transcript shared a large diurnal fluctuation under SC (Fig. 5, Table 1).

## Discussion

CAM shares a significant carbon-concentrating mechanism for aquatic plants in submerged environments and it is plastic in response to the environmental changes^11^,^14^,^17^,^28^. In this study, *I. sinensis* was used to elucidate the photosynthetic characteristics under SC and TC. Previous researches demonstrate that *I. sinensis* exhibits appreciable CAM photosynthesis under SC^33^. However, dynamic characteristics for the CAM photosynthesis in *I. sinensis* are poorly elucidated. In this article, we gradually investigated the photosynthetic characteristics during a diurnal cycle and further compared the characteristics under SC with TC. The results showed that titratable acidity levels and organic acid concentrations during a diurnal cycle were higher under SC than TC, indicating that *I. sinensis* possessed a stronger CAM capacity under SC than TC. When exposed to aerial environments, most species in *Isoetes* alternate the photosynthetic pathway from CAM to C_3_^11^,^15^. Yet, there are still quite a few species remaining CAM as the leaves are exposed to air, such as *I. karstenii, I. nuttallii* and *I. australis*^11^,^22^. Although the mechanism for CAM loss or reservation in *Isoetes* is still unknown, a widely acceptable opinion suggests that switching between CAM and C3 is to respond to the elevated CO_2_ in air, whereas the fixed CAM is to be a conservative strategy, considering that switching back and forth between C_3_ and CAM is of little benefit^11^,^15^,^22^. Titratable acidity and organic acid assays are popular diagnostic tests to detect the CAM activity^5^,^16^. Under TC, the total titratable acidity levels and malate concentrations were slightly higher in the night than the daytime, indicating that *I. sinensis* under TC shared a reduced CAM activity. Statistical data show that the titratable acidity levels from substantial CAM plants are variable from 5 to 300 μmolequiv g−1 FW^10^,^11^,^12^,^36^. Moreover, previous research declares that the acidity levels for *I. sinensis* under SC vary between 10.47 and 11.06 μmolequiv g−1 FW in the late afternoon and between 105.71 and 111.57 μmolequiv g−1 FW in the early morning^33^. In this article, our results revealed that the acidity levels under SC ranged from 22.44 to 29.28 μmolequiv g−1 FW in the late afternoon (from 15 to 18 h) and from 75.77 to 102.95 μmolequiv g−1 FW in the early morning (from 3 to 6 h). The results are understandable that CAM activity is variable depending on seasons and environments, such as pH, temperature, CO_2_, light level and ion concentrations^13^,^14^,^28^. Compared with under SC, the acidity levels under TC varied between 14.53 and 17.09 μmolequiv g−1 FW in the late afternoon and between 30.13 and 46.96 μmolequiv g−1 FW in the early morning, further revealing a decreased CAM activity under TC. Additionally, the malate concentration was decreased from 3 to 6 h in the early morning, speculating that *I. sinensis* probably brought forward to make preparations to satisfy the requirements in the following day. Citric acid is another carbon-fixed product in the night and occupies 10–20% dark-fixed components based on steady-state ^14^C labeling studies^10^,^11^. In this study, the citrate concentration showed a large diurnal amplitude under both conditions, suggesting that citric acid probably is not a suitable indicator to reflect the CAM activity in *Isoetes*^11^,^22^. But the malate and citrate concentrations consistently present obviously diurnal fluctuations in the typical CAM plant *Mesembryanthemum crystallinum*^15^,^37^.

Starch is an important product in the photosynthetic pathway and it is stored in leaves during the daytime and broken out in the following night^18^. Starch plays a pivotal role in serving as a carbon overflow to allow photosynthetic process faster and acting as a continuous carbon supply at night^35^,^38^. For the CAM plants, starch has an added role in providing the three-carbon CO_2_ acceptor molecule phosphoenolpyruvate in glycolysis pathway^39^. Moreover, starch metabolism is plastic to adapt to the environmental changes in terrestrial and aquatic plants^18^,^40^,^41^. In this study, the starch and soluble sugar levels were higher under TC than SC. Furthermore, the starch levels during the daytime were increasing under both conditions. In the night, the amplitude of starch level was low under SC, whereas a large amplitude was observed under TC (Fig. 3). Although starch can provide the substrate for malic acid synthesis, the starch accumulation in leaves is generally not completely in accordance with the malate and acidity levels^18^. The fact further demonstrates that starch is transferred to the corm or translated into phosphoenolpyruvate at proper times^11^,^18^.

Nocturnal CO_2_ in the cytosol is captured by PEPC enzyme in the night^16^. However, the PEPC activity probably is not limited in the dark, considering the fact that the enzyme can enhance Calvin activity during the daytime^11^. The PEPC activity over a diurnal cycle was slightly higher under SC than TC and there was a statistically significant difference at 9,12 and 3 h by comparing the activity under TC with SC (Table 1). Moreover, the PEPC activity consistently decreased during the daytime and increased from 18 to 0 h under both conditions (Fig. 4). In addition, for the *PEPC* gene, the transcript was more enriched under SC than TC and there was a statistically significant difference at most of the times by comparing the expression levels under TC and SC (Fig. 5). Furthermore, the total *PEPC* expression levels were higher in the night than the daytime under both conditions. The discrepancy between mRNA levels and protein abundance is considered that changes in mRNA stability and translational efficiency are likely to regulate the genes’ expression in response to the environmental changes^42^,^43^. Moreover, *PEPC* probably has some paralogous members recruited in the family to fulfill the increased carbon flux demand for CAM photosynthesis^44^.

Rubisco enzyme not only plays critical roles in photosynthetic pathway but also it is functional in photorespiration metabolism. In this study, the total Rubisco activity during the daytime was higher under TC than SC and the activity under both conditions presented a statistically significant difference at 12 and 18 h (Table 1). An apparent midday depression for photosynthesis and high light intensity presumably contribute to the decreasing Rubisco activity from 12 to 15 h under TC. However, for the *Rubisco* transcript, the mRNA levels were greater under TC than SC at most of the times and there was a statistically significant difference at 6, 12 and 15 h by comparing the expression levels under TC with SC. Nonetheless, the *Rubisco* transcript was decreasing during the daytime and increasing in the night under SC. When exposed to under TC, the plants suffered greater photorespiration and light intensity than under SC, which partially have effect on the discrepancy between transcripts and protein activity. Furthermore, post-transcription modification, temporal translation, mRNA unstability, low-translation efficiency and other *Rubisco* homologues consistently contribute to the temporal difference between mRNA levels and protein activity^16^,^23^,^42^. Compared the total enzyme activity of Rubisco with PEPC, the Rubisco activity was from 2.24 to 9.2-fold higher than PEPC activity under SC and from 2.52 to 18.60-fold under TC (Supplementary Figure S3). Nevertheless, previous studies reveal that the Rubisco activities in *I. howellii, I. lacustrisis* and *I. orcuttii* are from 3.4 to 7.2-fold higher than PEPC activity under SC and from 30.7 to 32.0- fold changes under TC^11^. The dynamic enzyme activities in this study are reliable with the diurnal changes, further suggesting that enzyme activity is affected by species diversity and environmental factors^11^. *Isoetes* can absorb additional CO_2_ from sediments in order to enhance carbon pool^11^. More than 95% CO_2_ is captured from sediments when CO_2_ levels in water are decreased, whereas the root uptake probably drops into less than 50% as increased CO_2_ levels in water^11^. PEPC has a high CO_2_ affinity under SC, probably resulting from the fact that vacuole occupies a limited storage capacity for malic acid^11^. Additionally, amphibious species in *Isoetes* do not have functional stomata in submerged environments^34^. The behaviors of stomata with the environmental changes in *I. sinensis* should be further investigated in the future.

CAM photosynthesis is controlled not only by environmental factors but also by genes associated with the regulations of transcription, post–transcription and post–translation^13^. To date, CAM research is mainly limited to biochemistry and physiological aspects. Molecular resources are very limited in public database. High-throughput sequencing approach becomes a perfect alternative for non-model plants to produce multiple sequencing data at a relatively low cost^45^,^46^,^47^,^48^. Furthermore, RNA-sequencing technology has become popular to discover novel genes and explore different expression profiles under various conditions^46^,^47^. In this study, we used RNA-sequencing to excavate photosynthetic genes in *I. sinensis*. In total, nine homologous genes were identified, including *PEPC*, *PPCK*, *MDH*, *Rubisco*, *GADPH*, *GAPA*, *PRK*, *PGK* and *V-ATPase*. Furthermore, we applied qPCR to describe expression profiles from the harvested materials under TC and SC, respectively. In general, the C_3_-related transcripts were enriched under TC, including the genes *Rubisco*, *PGK*, *GAPDH* and *GAPA*, whereas CAM-related transcripts were abundant under SC, including *PEPC*, *PPCK* and *MDH* transcripts. We further plotted dynamic diagrams to compare the expression patterns under TC with SC (Supplementary Figure S4 and S5). Compared with under SC, the transcripts showed a larger fluctuation under TC, probably indicating that the plants attempt to respond to the external stresses in terrestrial environments with high light intensity, limited water and CO_2_. In summary, investigation for the photosynthetic genes might be a breakthrough improvement to explore CAM pathway in *Isoetes* and it probably provides a novel insight into the study of CAM photosynthesis.

## Methods

### Plant materials and growth conditions

*I. sinensis* was collected from Xinan River with a fluctuated water level in Zhejiang Province, China (29°28′N; 119°14′E) and then cultivated in a greenhouse in Wuhan University. Initially, all experimental materials were maintained in submerged cultures with five plants in one growth chamber (30 × 40 × 50 cm), containing soil and 60 L tap water. We randomly chose a part of experimental materials to still keep the plants under SC with the water about 4 cm above the tops of leaves. The others were transferred to TC with leaves and shoots fully in air for an additional month. During the experimental period, weeds or snails were removed every day to maintain the experimental materials as a monoculture. Temperatures in water and air were recorded every three hours during a diurnal cycle. Juvenile and green leaves under SC and TC were collected over a diurnal cycle, respectively, including at 9, 12 15 and 18 h during the daytime and at 21, 0, 3 and 6 h in the night. All the materials were harvested in triplicate. Then the materials were washed in distilled water, immediately frozen into liquid nitrogen and stored at −80 °C, respectively.

### Titratable acidity assay

Diurnal fluctuation in titratable acidity for tissue extracts is considered to be a diagnostic test for CAM activity^28^,^49^. In this study, we employed a modified protocol to measure the titratable acidity^17^. Briefly, about 0.2 g frozen samples were immediately blotted dry, carefully weighed, and ground using a mortar and pestle on ice. Then 10 mL CO_2_-free distilled water was added to the mashed mixtures and further transferred to a plastic stopper tube. The solutions were boiled for 30 min and cooled to room temperature (25 °C). Finally, the mixtures were titrated to pH 8.3 using 0.01 mol L^−1^ NaOH, which was standardized by Gran titration against 0.1 mol L^−1^ HCl^17^,^22^,^50^. The experiments were carried out in triplicate and the results were described as μmolequiv g^−1^ FW.

### Organic acid determination

Diurnal fluctuations in malate and citrate concentrations were determined following the procedure of Pedersen^22^. About 0.4 g collected materials were ground using a cold mortar and pestle on ice, and plunged in ice–cold 5% perchloric acid^51^. The mixture was centrifuged at 12000 g for 20 min and the supernatant was collected. Then the remnants were extracted again using ice–cold 5% perchloric acid. The supernatants were combined, adjusted pH to 3.5 using K_2_CO_3_, and then centrifuged at 12000 g for 10 min. Finally, the supernatants were collected, filtered (0.22 μm) and stored at 4 °C before HPLC analysis.

Malate and citrate in the leaf extracts were detected by HPLC with a 600 E pump, 717 plus autoinjector and 996 Photodiade array detector (Waters, Milford, MA, USA). In brief, isolates were obtained at 22 ± 0.5 °C on a C_18_ column (250*4.6 mm internal diameter with 5-μm packing; Alltech Associates, Deerfield, IL, USA). A mobile phase was composed of 25 mM KH_2_PO_4_ (pH 2.5), concentrated perchloric acid and 60% methanol with the flow speed of 1 mL min^−1^. All data were obtained and analyzed by EMPOWER^®^ chromatography software (Waters). Photo Diode Array (PDA) at 210 nm was used to determine and quantify the tested compounds. Malate and citrate Standards (Sigma St Louis, MO, USA) were used to identify the effect on spectral characteristics, retention time and PDA peak spectral analysis. All materials in this experiments were measured in triplicate.

### Starch and soluble sugar measurements

The levels of starch and soluble sugar were measured using colorimetric metho^52^. In brief, 0.4 g fresh materials were pulverized in a mortar using liquid nitrogen. 10 mL distilled water was added into the mashed materials and then transferred to a plastic stopper tube. We boiled the tube for 20 min and added 15 mL distilled water to the tube. Initially, the amounts of sugar or soluble sugar were graded from 0 to 10 μg and the absorbance was measured at 485 nm. Then calibration curves were plotted in order to quantify the experimental materials. For the soluble sugar tests, 0.5 mL supernatants were added into a mixture solution with 1mL 9% phenol and 5 mL concentrated sulfuric acid. After 30 minutes, the absorbance was recorded at 485 nm in triplicate.

The remnants after extracting soluble sugar were further used to separate starch using colorimetric method^52^. 20 mL distilled water was added into the tubes and further boiled for 15 min. Then 9.2 mol L^−1^ perchloric acid was added into the cooled solution for 15 min. The mixtures were further filtered (0.22 μm) and centrifuged at 12000 g for 10 min. We initially used a I_2_-KI process (0.06g I_2_ and 0.6 g KI in 100 ml distilled water with 0.05 M HCl) to detect starch in the extracts. 1 mL I_2_-KI mixture was added into 100 μL supernatants and the solutions were maintained at room temperature for 10 min. Then a total of 1 mL 9% phenol and 5 mL concentrated sulfuric acid were mixed with the extracts for 30 min at room temperature. Finally, the absorbance was detected at 485 nm in triplicate.

### Soluble protein and enzyme activity measurements

Instructions for the extraction and measurement of PEPC and Rubisco enzyme activity were slightly adapting to the protocols described by Yizhi Zhang^50^. Briefly, 0.5 g harvested materials were blotted dry and immediately weighed to guarantee the fresh weight (FW). The weighted samples were then pulverized in a cold mortar using liquid nitrogen, mixed with 3 mL extraction buffer, transferred to a plastic stopper tube and centrifuged at 4 °C for 15 min at 12000 g. The extraction buffer included 50 mM Tris, 15 mM MgCl_2_, 0.1 mM EDTA, and 10% glycerol (pH 8.0). The crude extracts were stored at 4 °C and measured within an hour. Initially, soluble protein in the crude extracts was assayed spectrophotometrically (UV-2600, UNICO) using bovine serum albumin as a standard sample^53^. The Rubisco activity was measured in a reaction mixture containing 50 mM Tris, 15 mM MgCl_2_, 0.1 mM EDTA, 20 mM bicarbonate, 0.2 mM NADH (Sigma), 5 mM DTT, 1 mM ATP, 5 units of glyceraldehyde-3-phosphate dehydrogenase from rabbit muscle (GAPDH; Sigma), 5 units of phosphoglycerate kinase from yeast (PGK; Sigma) and 1 mM ribulose 1, 5-bisphosphate (RUBP; Sigma). The enzyme reaction was initiated by adding 20 μL extracts to the mixtures and the absorbance at 340 nm was calculated after 5 min at 25 °C. In the meantime, the calculated Rubisco activity should take into consideration that two NADH molecules are catalyzed by one RUBP molecule. In addition, PEPC activity in the crude extracts was measured in a reaction mixture, including 50 mM Tris, 15 mM MgCl_2_, 0.1 mM EDTA, 20 mM bicarbonate, 0.2 mM NADH and 5 units of malate dehydrogenase (MDH; Sigma), 0.2 mM NADH and 1 mM phosphoenol pyruvate (Sigma). The reaction was initiated by supplying 30 μL extracts and the absorbance at 340 nm was recorded after 5min at 25 °C. In this study, R program was employed for two-way ANOVA with Tukey *post hoc* test and further used to compare means^54^. P-value less than 0.01 was considered to have a statistically significant difference. In addition, gglpot2 package in the R program was used to plot graphics in this study^55^.

### Identification of photosynthetic genes based on RNA-sequencing technology

We applied RNA-sequencing technology to obtain photosynthetic genes in *Isoetes*. Briefly, juvenile leaves were harvested under TC and SC, respectively. Then the leaves were immediately frozen in liquid nitrogen and stored at –80°C prior to RNA extraction. Total RNA was extracted using Trizol (Invitrogen Inc., USA) and residual DNA was further removed with RNase-free DNase I (Takara, Da Lian, China) according to the manufacturers’ instruction. The RNA quality and quantity were verified using 2100 Bioanalyzer RNA Nanochip (Aligent, CA, USA) and ND-1000 Nanodrop Spectrophotometer (Thermo scientific, DE, USA), respectively. Equal amounts of total RNA were mixed from three independent extractions to a single combined sample. The mRNA was purified by oligo(dT) magnetic beads, fragmented to about 200 bp, and further synthesized into first-strand cDNA using random hexamer primers and reverse transcriptase. Then second-strand cDNA was synthesized using RHase H (New England Biolabs Inc., Ipswich, MA, USA) and DNA polymerase (Invitrogen, Carlsbad, CA, USA). We further repaired the end fragments and ligated them with sequencing adaptors. Appropriate fragment ranges for PCR application (200±25bp) were selected by agarose gel electrophoresis and purified using a QiaQuick PCR extraction kit (Qiagen, USA). Finally, the cDNA libraries were sequenced on an Illumina Hiseq 2000 platform to generate 100 pair-end raw reads, according to the manufacturers’ recommendation (Illumina Inc., San Diego, CA, USA).

The raw reads initially conducted a general quality control analysis using FastQC^56^. The filtering thresholds included removing adaptors, ambiguous nucleotide reads with N, polymerase chain reaction artifacts, and low quality bases with an average Phred score less than 20. After filtering and trimming the contaminations and bad quality sequences, all clean reads were merged and further de novo assembled into unigenes using Trinity program^57^, setting k-means length to 25. All unigenes were further searched using BLASTx against the non-redundant database with an E-value cutoff of 10^−5^. For the sequences failed to be searched in the database, we used GetORF software to predict their orientations and underlying protein coding regions^58^.

### Transcript accumulations of photosynthetic genes using qPCR

Approximately 0.1 g harvested materials were immediately frozen by liquid nitrogen in a cold mortar. Total RNA was extracted using RNAiso Plus (Takara, Da Lian, China), according to the manufacturers’ instruction. The extracted RNA was incubated using 1 μL RNase-free DNase I (Promega, Madison, WI, USA) for 15 min at 37 °C to eliminate genomic DNA. Then 1μg total RNA was reverse transcribed into single-strand cDNA using Primerscript^TM^ One Step RT-PCR Kit Ver.2 following the manufacturers’ protocol (Takara, Da Lian, China). Gene-specific primers were designed using a free online primer design tool (http://primer3plus.com/cgi-bin/dev/primer3plus.cgi). The qPCR analysis was performed on a CFX96 Real-time PCR system (Bio-Rad, Hercules, USA). The cDNA initially was diluted ten-fold and used as templates in the qPCR tests. The reaction mixtures (25 μL) included 0.25 pmol forward and reverse primers, 12.5 μL 2 × SYBR premix (Takara, Da Lian, China), 2.5 μL diluted cDNA, and 7.0 μL sterile water. The PCR program was an initial denaturation step of 3 min at 95 °C, followed by 40 cycles of 95 °C for 30 s, annealing temperatures for 15 s and finally 72 °C for 30 s. Specific annealing temperature and related information for the photosynthetic genes are listed in Supplementary Table S1. The relative expression levels were calculated using 2^−△△Ct^ method^59^, and normalized to the geometric average of Ct values with the *actin* gene as an internal control. All experiments and analysis were performed in triplicate.

## Additional Information

**How to cite this article**: Yang, T. and Liu, X. Comparing photosynthetic characteristics of *Isoetes sinensis* Pamler under submerged and terrestrial conditions. *Sci. Rep.* **5**, 17783; doi: 10.1038/srep17783 (2015).

## Supplementary Material

Supplementary Information

## Figures and Tables

**Table 1:** Diurnal fluctuation of the levels of temperature, titratable acidity, malate, citrate, starch, soluble sugar, soluble protein, PEPC activity, Rubisco activity and relative expression levels of nine photosynthetic genes under submerged (SC) and terrestrial conditions (TC).

Terrestrial	Hour (h)							
	6	9	12	15	18	21	0	3
Temperature(°C)	14.23 ± 0.50	25.03 ± 0.26^a^	25.10 ± 0.2^a^	27.10 ± 0.21^a^	23.03 ± 0.26^a^	20.10 ± 0.32	14.13 ± 0.30	14.10 ± 0.21
Acidity(μmolequiv g−1 FW)	46.96 ± 1.98^a^	31.75 ± 1.59	12.56 ± 1.8^a^	17.09 ± 2.72	14.53 ± 2.2 5^a^	26.90 ± 2.29	39.18 ± 4.39^a^	30.13 ± 2.95^a^
Malate(mg. g^−1^ FW)	34.01 ± 1.79^a^	26.18 ± 2.36^a^	23.35 ± 1.71^a^	23.30 ± 1.67^a^	21.83 ± 1.50^a^	30.97 ± 1.49^a^	41.19 ± 2.57^a^	49.68 ± 2.95^a^
Citrate(mg. g^−1^ FW)	35.30 ± 2.08	26.02 ± 2.33	27.30 ± 2.09^a^	28.56 ± 1.73	27.48 ± 2.01	19.54 ± 2.37^a^	27.09 ± 1.63	30.01 ± 1.6 3^a^
Starch(%)	0.75 ± 0.01	0.80 ± 0.01^a^	0.94 ± 0.03	0.86 ± 0.02	1.42 ± 0.06^a^	1.03 ± 0.04	0.93 ± 0.02	1.25 ± 0.01^a^
Soluble sugar(mg/g)	14.23 ± 0.38^a^	68.61 ± 0.78^a^	16.97 ± 0.28^a^	53.35 ± 1.06^a^	14.56 ± 0.25	50.28 ± 1.21^a^	23.52 ± 0.25^a^	38.26 ± 0.41^a^
Soluble protein(μg/g)	2.91 ± 0.05^a^	2.04 ± 0.04^a^	1.30 ± 0.01^a^	2.58 ± 0.02^a^	2.29 ± 0.11	2.39 ± 0.0 4^a^	1.85 ± 0.05	2.86 ± 0.20^a^
PEPC activity(μmol mg^−1^ protein min^−1^)	3.66 ± 0.45	2.16 ± 0.18	2.08 ± 0.23^a^	2.28 ± 0.27	2.11 ± 0.15	3.85 ± 0.09	4.69 ± 0.23	4.08 ± 0.18^a^
Rubisco activity(μmol mg^−1^ protein min^−1^)	12.78 ± 0.67	19.69 ± 0.90	37.60 ± 1.43^a^	25.64 ± 1.32	30.84 ± 1.19^a^	12.72 ± 0.46	12.60 ± 0.86	10.01 ± 0.55^a^
* PEPC*	0.16 ± 0.05^a^	0.16 ± 0.04	0.09 ± 0.02	0.02 ± 0.01^a^	0.14 ± 0.11^a^	0.22 ± 0.05^a^	0.31 ± 0.06^a^	0.10 ± 0.0 8^a^
* PPCK*	0.21 ± 0.01^a^	0.37 ± 0.04	0.28 ± 0.02^a^	0.07 ± 0.01^a^	0.12 ± 0.07^a^	0.66 ± 0.02	0.35 ± 0.19	0.29 ± 0.04
* MDH*	0.31 ± 0.05	0.54 ± 0.01	0.68 ± 0.00	0.14 ± 0.02^a^	0.21 ± 0.01^a^	0.46 ± 0.00	0.02 ± 0.10	0.15 ± 0.05
* PPCK*	5.28 ± 0.67^a^	1.35 ± 0.20	10.31 ± 0.05^a^	2.28 ± 0.34	8.35 ± 0.54	2.50 ± 0.90	2.12 ± 0.00	1.59 ± 0.30
* Rubisco*	1.00 ± 0.16^a^	0.37 ± 0.06	0.56 ± 0.07^a^	0.56 ± 0.05^a^	0.47 ± 0.05	0.59 ± 0.07	0.58 ± 0.05	0.69 ± 0.06
* GAPDH*	1.72 ± 0.12^a^	0.02 ± 0.04	0.40 ± 0.02	0.13 ± 0.01	0.21 ± 0.08	0.40 ± 0.01	0.24 ± 0.02	0.35 ± 0.05
* GAPA*	2.11 ± 0.01^a^	0.03 ± 0.04	0.38 ± 0.01	0.16 ± 0.06	0.29 ± 0.02	0.41 ± 0.00	0.31 ± 0.04	0.30 ± 0.08
* PGK*	0.98 ± 0.03	1.01 ± 0.02	1.23 ± 0.03	8.00 ± 0.21^a^	1.42 ± 0.03	3.09 ± 0.55^a^	3.40 ± 0.08	0.87 ± 0.09
* V-ATPase*	0.25 ± 0.04^a^	0.31 ± 0.02	0.50 ± 0.18	0.63 ± 0.03	0.47 ± 0.18	0.23 ± 0.04	0.11 ± 0.01	0.34 ± 0.05
Submerged								
Temperature(°C)	14.10 ± 0.26	23.10 ± 0.21^a^	23.03 ± 0.20^a^	20.03 ± 0.15^a^	20.03 ± 0.15^a^	20.00 ± 0.00	14.07 ± 0.12	14.00 ± 0.12
Acidity(μmolequiv g−1 FW)	102.95 ± 1.50^a^	36.79 ± 1.00	34.45 ± 0.59^a^	22.44 ± 2.35	29.28 ± 1.29^a^	39.06 ± 2.63	56.33 ± 3.08^a^	75.77 ± 1.06^a^
Malate(mg. g^−1^ FW)	93.43 ± 3.99^a^	84.17 ± 2.54^a^	67.42 ± 1.68^a^	62.52 ± 2.19^a^	60.70 ± 2.20^a^	72.70 ± 3.13^a^	79.29 ± 3.28^a^	180.93 ± 5.10^a^
Citrate(mg. g^−1^ FW)	40.94 ± 1.66	32.01 ± 1.76	52.44 ± 2.12^a^	32.21 ± 1.27	31.04 ± 2.15	59.28 ± 2.74^a^	29.44 ± 1.07	57.30 ± 2.26^a^
Starch(%)	0.26 ± 0.03	0.27 ± 0.00^a^	0.66 ± 0.03	0.78 ± 0.02	0.73 ± 0.02^a^	0.61 ± 0.01	0.54 ± 0.00	0.22 ± 0.32^a^
Soluble sugar(mg/g)	6.88 ± 0.12^a^	26.43 ± 1.06^a^	4.47 ± 0.6 4^a^	8.34 ± 0.28^a^	11.73 ± 1.52	12.50 ± 0.78^a^	4.38 ± 0.15^a^	22.20 ± 1.20^a^
Soluble protein(μg/g)	1.48 ± 0.13^a^	1.26 ± 0.09^a^	1.87 ± 0.04^a^	1.73 ± 0.00^a^	2.14 ± 0.04	1.71 ± 0.12^a^	2.04 ± 0.03	1.67 ± 0.04^a^
PEPC activity(μmol mg^−1^ protein min^−1^)	4.69 ± 0.26	3.59 ± 0.18	3.43 ± 0.30^a^	2.43 ± 0.31	2.19 ± 0.24	4.16 ± 0.29	4.83 ± 0.19	5.95 ± 0.26^a^
Rubisco activity(μmol mg^−1^ protein min^−1^)	10.82 ± 0.55	14.95 ± 0.85	17.65 ± 1.19^a^	20.72 ± 0.56	19.76 ± 1.71^a^	18.75 ± 0.51	16.60 ± 0.82	18.66 ± 1.64^a^
*PEPC*	1.00 ± 0.09^a^	0.36 ± 0.02	0.17 ± 0.10	1.07 ± 0.06^a^	1.51 ± 0.04^a^	0.75 ± 0.08^a^	1.58 ± 0.02^a^	0.77 ± 0.03^a^
*PPCK*	1.00 ± 0.09^a^	0.73 ± 0.06	1.50 ± 0.05^a^	0.75 ± 0.07^a^	1.43 ± 0.11^a^	0.90 ± 0.11	0.98 ± 0.08	0.84 ± 0.04
*MDH*	1.00 ± 0.01	0.53 ± 0.01	0.45 ± 0.00	3.00 ± 0.02^a^	1.73 ± 0.01^a^	1.08 ± 0.03	0.02 ± 0.03	0.90 ± 0.01
*PPCK*	1.00 ± 0.53^a^	0.66 ± 0.14	2.60 ± 0.70^a^	3.14 ± 0.55	5.90 ± 1.10	1.56 ± 0.08	1.12 ± 0.27	4.13 ± 0.35
*Rubisco*	1.00 ± 0.10^a^	0.37 ± 0.04	0.13 ± 0.04^a^	0.17 ± 0.02^a^	0.35 ± 0.02	0.35 ± 0.05	0.70 ± 0.05	0.71 ± 0.03
*GAPDH*	1.00 ± 0.02^a^	0.03 ± 0.04	0.001 ± 0.00	0.16 ± 0.02	0.06 ± 0.02	0.26 ± 0.03	0.07 ± 0.07	0.25 ± 0.04
*GAPA*	1.00 ± 0.03^a^	0.03 ± 0.02	0.002 ± 0.11	0.20 ± 0.03	0.06 ± 0.03	0.26 ± 0.00	0.18 ± 0.18	0.20 ± 0.05
*PGK*	1.00 ± 0.03	0.82 ± 0.14	0.82 ± 0.04	2.90 ± 0.13^a^	0.43 ± 0.10	15.58 ± 0.32^a^	2.06 ± 0.07	1.05 ± 0.07
*V-ATPase*	1.00 ± 0.32^a^	0.40 ± 0.05	0.65 ± 0.36	0.56 ± 0.04	0.13 ± 0.04	0.34 ± 0.01	0.62 ± 0.06	0.50 ± 0.09

Juvenile and green leaves were harvested every three hours during the daytime (from 6 to 18 h) and night (from 18 to 6 h) under TC and SC, respectively. Letters indicate statistically significant differences (ANOVA and Tukey *post hoc* test; P < 0.01) between the levels under TC and SC. Numbers indicate the mean ± SE (n = 3).
