# Supplementary material for: Comparing photosynthetic characteristics of Isoetes sinensis Palmer under submerged and terrestrial conditions
Source: Sci Rep. 2015 Dec 4;5:17783. doi: 10.1038/srep17783 (PMC4669503; doi:10.1038/srep17783)
Supplement: Supplementary Information [file srep17783-s1.pdf]

Comparing photosynthetic characteristics of *Isoetes sinensis* Palmer under submerged and terrestrial conditions

Tao Yang [1], Xing Liu [1,*]

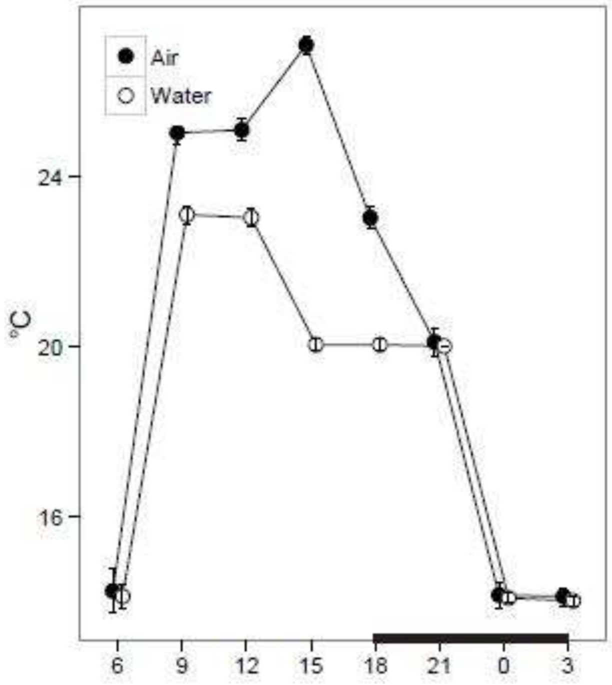

Supplementary Figure S1. Diurnal fluctuation of the temperatures in water and air.

Temperatures in water and air were recorded during the daytime (white) and night (black), respectively.

Each point represents the mean ± SE (n=3).

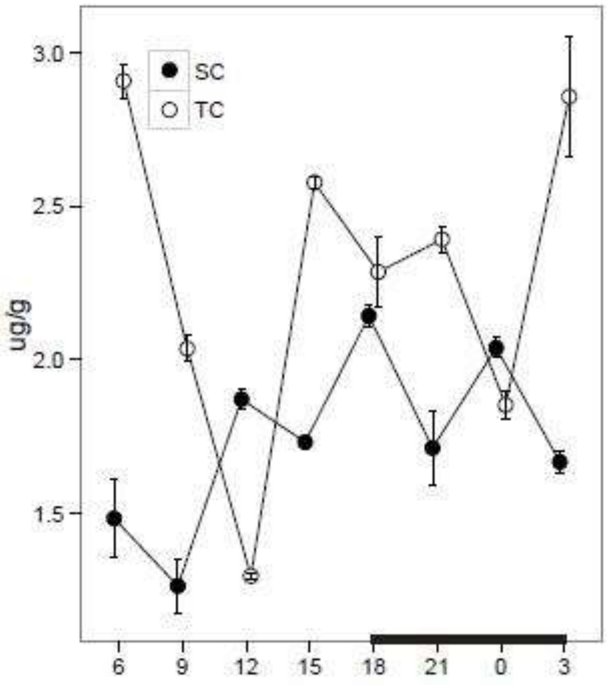

Supplementary Figure S2. Diurnal fluctuation of the levels of soluble protein under submerged (SC) and terrestrial conditions (TC).

Juvenile and green leaves were harvested every three hours during the daytime (white) and night (black), under TC and SC, respectively. Each point represents the mean ±SE (n=3).

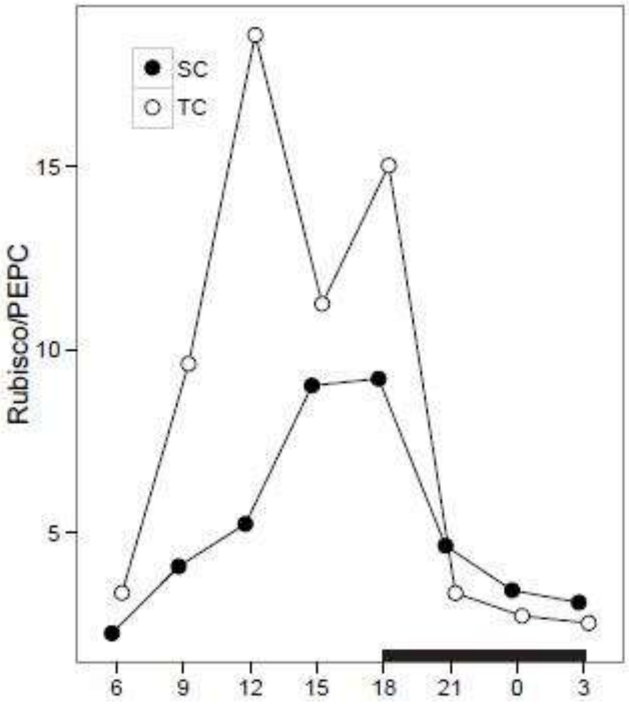

Supplementary Figure S3. Ratios of Rubisico and PEPC activity over a diurnal cycle under submerged (SC) and terrestrial conditions (TC).

Juvenile and green leaves were harvested every three hours during the daytime (white) and night (black), under TC and SC, respectively.

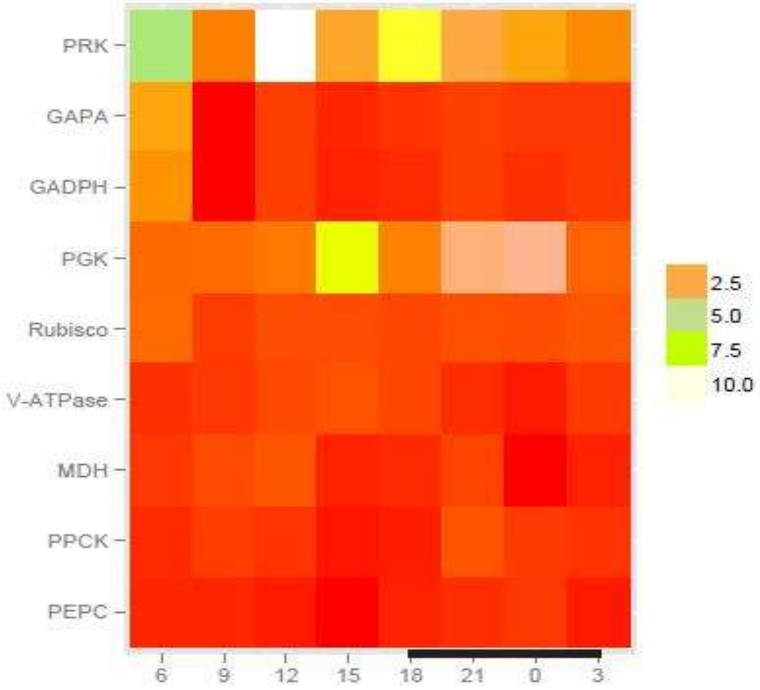

Supplementary Figure S4. Dynamic plot about the relative expression levels of nine photosynthetic genes under TC.

Juvenile and green leaves were harvested every three hours during the daytime (white) and night (black), respectively. Nine genes were associated with CAM photosynthesis, including *PEPC*, *PPCK* and *MDH* for CAM mode; *Rubisco*, *GADPH*, *GAPA*, *PRK* and *PGK* for C_3 mode; and *V-ATPase* associated with malic acid accumulation via the electrochemical channel of tonoplast. The legend in the right represents the relative expression levels and describes with different colors.

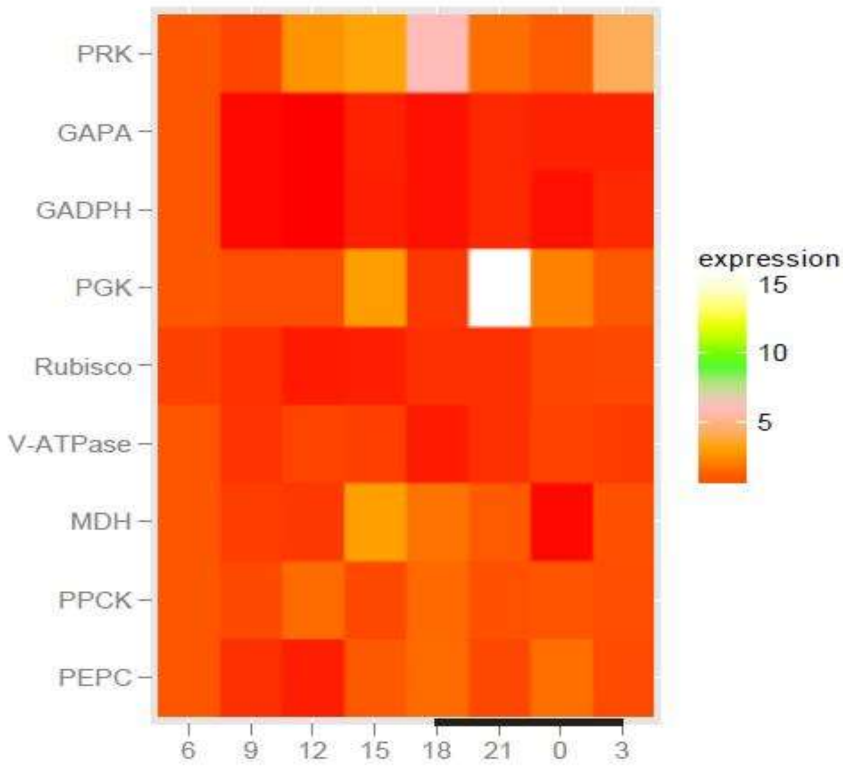

Supplementary Figure S5. Dynamic plot about the relative expression levels of nine photosynthetic genes under SC.

Juvenile and green leaves were harvested every three hours during the daytime (white) and night (black), respectively. Nine genes were associated with CAM photosynthesis, including *PEPC*, *PPCK* and *MDH* for CAM mode; *Rubisco*, *GADPH*, *GAPA*, *PRK* and *PGK* for C_3 mode; and *V-ATPase* associated with malic acid accumulation via the electrochemical channel of tonoplast. The legend in the right represents the relative expression levels and describes with different colors.

Genes	Forward primers	Reverse primers	Annealing temperatures (℃)	Annotations
PEPC	CAGTAGGAGGGGTCGGTCA	TGTTGCCTTGATGGGGACA	60	Phosphoenolpyruvate carboxylase
PPCK	TACATTGTCTGAGCAAGGGTG	CAGGTTTCTGTTCCGAGGC	60	Phosphoenolpyruvate carboxylase kinase
MDH	TCCCTTTCTTCCTTGAGC	CAGATGGGTCCTATGGTGT	54	Malic acid dehydrogenase
Rubisco	CCAATGATGCGAACGAAG	GCCCTGGATACTATGATGGAC	54	Ribulose-1, 5-carboxlyase
PGK	CAGTTGTACGGAATTGAGG	AAGAAGGGAAGAGTTATGGA	57	Phosphaglycerate kinase
GAPDH	ACCACCAAATCTACAACCG	ACCAACATCTACAGGAGCAG	54	glyceraldehyde 3-phosphate dehydrogenase
GAPA	GGCAAAGGTCTTCTTCTCGG	TCGGCATCGTCAAGGGTCT	52	glyceraldehyde-3-phosphate dehydrogenase (NADP+)
PRK	GTACGCCGACATGACCATTC	GGGTCCGTAGAAGAACTTGATT	54	Phosphoribulokinase
V-ATPase	GGTGCTGCGATGTATGAAC	CCATCAATCCTGCTGTTTC	57	H^+-translocation ATPase
actin	TCCTCTTCCAGCCTTCTTT	TTCCTCCACTGAGCACAATA	52 to 60	actin

Supplementary Table S1. Primers' information for qPCR analysis

Nine genes were associated with CAM photosynthesis, including *PEPC*, *PPCK* and *MDH* for CAM mode; *Rubisco*, *GADPH*, *GAPA*, *PRK* and *PGK* for C_3 mode; and *V-ATPase* associated with the malic acid accumulation via an electrochemical channel of tonoplast. The *actin* gene acts as an internal control in this study.
